# Patterns and dynamics of rapid local adaptation and sex in varying habitat types in rotifers

**DOI:** 10.1002/ece3.781

**Published:** 2013-10-01

**Authors:** Thomas Scheuerl, Claus-Peter Stelzer

**Affiliations:** 1 Imperial College LondonSilwood Park Campus, Berkshire, SL5 7PY, U.K; 2 Research Institute for Limnology, University of Innsbruck5310, Mondsee, Austria

**Keywords:** *Brachionus calyciflorus*, diapausing eggs, laboratory natural selection, local adaptation, rotifers, sex

## Abstract

Local adaptation is an important principle in a world of environmental change and might be critical for species persistence. We tested the hypothesis that replicated populations can attain rapid local adaptation under two varying laboratory environments. Clonal subpopulations of the cyclically parthenogenetic rotifer *Brachionus calyciflorus* were allowed to adapt to two varying harsh and a benign environment: a high-salt, a food-limited environment and untreated culture medium (no salt addition, high food). In contrast to most previous studies, we re-adjusted rotifer density to a fixed value (two individuals per ml) every 3–4 days of unrestricted population growth, instead of exchanging a fixed proportion of the culture medium. Thus our dilution regime specifically selected for high population growth during the entire experiment and it allowed us to continuously track changes in fitness (i.e., maximum population growth under the prevailing conditions) in each population. After 56 days (43 asexual and eight sexual generations) of selection, the populations in the harsh environments showed a significant increase in fitness over time relative to the beginning compared to the population in untreated culture medium. Furthermore, the high-salt population exhibited a significantly elevated ratio of sexual offspring from the start of the experiment, which suggested that this environment either triggered higher rates of sex or that the untreated medium and the food-limited environment suppressed sex. In a following assay of local adaptation we measured population fitness under “local” versus “foreign” conditions (populations adapted to this environment compared to those of the other environment) for both harsh habitats. We found significantly higher fitness values for the local populations (on average, a 38% higher fitness) compared to the foreign populations. Overall, local adaptation was formed rapidly and it seemed to be more pronounced in the high-salt treatment.

## Introduction

In recent research rotifers have been established as a model system for eco-evolutionary questions (Yoshida et al. 2003; Becks and Agrawal 2010; Becks et al. 2012). For example, they have been successfully used to investigate the role of sex during adaptation (Becks and Agrawal 2012). Demonstrating adaptation in rotifers is difficult, since cryopreservation, that is, the possibility of comparison with a frozen ancestor, is not well established (King et al. 1983; Toledo et al. 1991). If cryopreservation techniques are not available, adaptation in experimental lines may be measured by following the increase of mean population fitness or of offspring over time (Becks and Agrawal 2012). However, it is difficult to estimate whether a single general high fitness clone evolved due to laboratory selection, or if specific adaptation is observed selecting for two or more different clones. This could be estimated by tests of local adaptation (Kawecki and Ebert 2004; Blanquart et al. 2013). So far, for rotifers under laboratory selection local adaptation was not shown in detail. Local adaptation was shown for natural populations (Campillo et al. 2011; Alcántara-Rodríguez et al. 2012), but true local adaptation has not been shown so far for laboratory selected populations. Some authors did tests of local adaptation in laboratory rotifer populations, but the requirements for local adaptation were only partly fulfilled (Becks and Agrawal 2010). As rotifers are used as important model organisms in laboratory natural selection experiments it seems to be important to get further insight into to process of adapation in such populations. Our purpose of this study was to show local adaptation in rotifers and track changes in fitness directly in the populations.

Local adaptation, which is adaptation to specific environental conditions, can be estimated from measurements of performance (fitness) of populations adapted to varying habitats (Kawecki and Ebert 2004; Hereford 2009). Organisms in their “home” habitat should have a higher fitness compared to conditions in an “away” habitat, or more precisely, in the habitat of the other population (Kawecki and Ebert 2004). Furthermore, the organisms of the “local” habitat (their home habitat) should have a higher fitness compared to organisms from a “foreign” habitat (Kawecki and Ebert 2004). Only if both criteria of “home versus away” and “local versus foreign” are fullfilled, local adaptation is confirmed (Kawecki and Ebert 2004).

Selection regimes in the laboratory can be categorized as laboratory natural selection (LNS) or artificial selection (Garland and Rose 2009). While LNS aims at selection of performance (fitness) and allows multiple solutions for optimisation, artificial selection aims at selection of specific traits, for example, bristle number in *Drosophila* (Garland and Rose 2009). Examples of LNS in the literature include experiments demonstrating adaptation to a harsh and a benign environment in rotifers (Becks and Agrawal 2012), or to two harsh conditions in yeast (Gray and Goddard 2012a).

Local adaptation was investigated for many different organisms and is a highly important pattern observed in natural and laboratory systems (Hereford 2009). In our study we used the monogonont rotifer *Brachionus calyciflorus* to test for local adaptation after LNS. An open question we wanted to answer is whether rotifers can develop local adaptation under LNS in a minimum of time, where both criteria of “home versus away” and “local versus foreign” are fulfilled. This time was estimated by following the change of fitness directly in the populations. Thus, our study tries to combine both, the process of selection and adaptation over time to estimate divergent selection and the test of local adaptation for two different environments. A similar study was done by Gray and Goddard (2012a) using yeast, but, to our best knowledge, not for metazoans like rotifers. Furthermore, in contrast to previous studies, which had measured the fitness of individuals extracted from the experimental populations at specific time points (Becks and Agrawal 2012; Gray and Goddard 2012a), we wanted to follow the process of adaptation continuously in the experimental rotifer populations. Thus we re-adjusted rotifer density to a fixed value (two individuals per ml) every 3–4 days, instead of exchanging a fixed proportion of the culture medium (which is standard practice in such experiments). This allowed us to obtain an estimate of population growth for each transfer interval. In addition, our experimental design ensured that we always selected for maximum growth rate rather than tolerance of high population density. The latter is an inevitable consequence of dilution regimes that involve an exchange of a fixed proportion of the culture volume (Flegr 1997).

Monogonont rotifers are cyclical parthenogens with haploid dwarf males (Fussmann 2011). Females normally reproduce by ameiotic parthenogenesis, but sporadically undergo sexual reproduction (Nogrady et al. 1993). Induction of sexuality and production of males and diapausing eggs (embryos waiting in a resting stage) is mainly density dependent and triggered by a mixis inducing protein (Gilbert 2003a; Stelzer and Snell 2003; Schröder 2005; Snell et al. 2006).

We tested the hypothesis that these small metazoans can rapidly build up local adaptation when faced with two different laboratory environments: a food-limited environment, and a high-salt environment (oceanic sea salt). As soon as fitness (population growth) of the diverging populations appeared to reflect adaptation to the new conditions, we tested in a separate assay for fitness of “local” populations versus “foreign” populations and compared “home” versus “away” conditions. In this assay, fitness of single females was quantified as the ability to produce the highest number of offspring per unit time, which corresponds to selection for high population growth rates. To ensure that sexual offspring actually did recruit into our populations we additionally tested for spontaneous hatching of the resting eggs.

## Materials and Methods

### Isolation of diapausing eggs

Diapausing eggs from *Brachionus calyciflorus* were isolated from the sediments of a small lake called “Egelsee” in Upper-Austria using a sucrose flotation technique (García-Roger et al. 2006), but see Data S1 for further details of isolation. Embryos were incubated over night in COMBO medium (Kilham et al. 1998) with the algae *Chlamydomonas reinhardtii* as a food source (Strain: SAG11-32b, Sammlung fuer Algenkulturen, Goettingen, Germany). Hatchlings were collected during the following 3 days and were cultivated as monoclonal stem cultures for 3 weeks to allow acclimatization to laboratory conditions (see Data S1).

### Spontaneous hatching rate

We estimated rates of spontaneous hatching for diapausing eggs in order to test if recombinant clones could hatch and contribute to the populations in our selection and adaptation experiments. This was done by incubating diapausing eggs in COMBO medium directly after production versus after a cold and dark period of 14 days at 8°C (see Data S1). In total seven 48-well-plates (336 eggs, 1 per well) were directly incubated at 23°C to test for spontaneous hatching (“Light” treatment). Another seven 48-well-plates (336 eggs) were covered with aluminum foil and stored for 14 days at 8°C in the dark (“Dark” treatment). Hatching was recorded over a time period of 9 days at daily intervals for both treatments after incubation at room temperature with algae.

### Divergent selection

To test whether a population of *B. calyciflorus* rotifers is able to adapt to varying laboratory conditions, two treatments reducing the fitness of rotifers were established with *C. reinhardtii* as food. Populations in 1 L aerated bottles were transferred to fresh food conditions twice per week spaced in two intervals, a long period of 4 days and a short period of 3 days. We used two modifications of COMBO medium, which were empirically determined in pilot experiments (see Data S1). First, we used a high-salt medium (SM) for producing high-salt populations (SP). This was obtained by adding an unnaturally high concentration of 5.5 g/L salt (oceanic sea salt) to the medium (360 μmol N and 300,000 algae cells/mL). Second, in a food-limited medium (FM) we used a reduced algae and nitrogen concentration producing food-limited populations (FP). The growth of the algae was inhibited by a nitrogen content of 5 μmol N for the short period and 10 μmol N for the long period in this treatment, in contrast to 360 μmol/L N which are used under standard conditions. Additionally the food level was restricted to lower concentrations of *C*. *reinhardtii* (150,000 cells/mL for the short period & 180,000 cells/mL for the long period). Continuous illumination was provided with daylight fluorescent bulbs (30–40 μEinstein/m^2^/sec). We used five replicate populations for each treatment.

For the initial inoculation of each replicate population, 83 isolated clones of *B. calyciflorus* were used and maintained in monoclonal cultures. Three weeks after hatching, the clones were pre-cultivated under low-density conditions for 4 days. Each clone reproduced asexually during this time, which allowed us to inoculate between 15 and 25 females of each clone into each replicate population. The populations of the FM and SM treatments were acclimatized to the stressful conditions by a reduced selection-step for the first 3 days (FM: 10 μmol N and 180,000 cells/mL and SM: 5 g/L).

During the experiment populations were re-inoculated every 3–4 days in an alternating rhythm. The transferred population sizes were always 2000 individuals per 900 mL (2.2 females/mL). The population density of females at the end of each 3–4 days growth period was used to calculate the culture volume that was needed for a new inoculum of 2000 females. Initial and final densities were used to calculate the population growth rate *r*, our measure of population fitness. Counts of the population density were based on the mean of three independent samples. Additionally the same clonal subpopulations of rotifers were cultivated in normal-COMBO medium (CM, 360 μmol N and 300,000 algae cells/mL) establishing COMBO-Populations (CP) to test whether fitness was indeed reduced by the selection treatments.

Our method removed the mixis inducing protein (MIP) every 3 or 4 days. Theoretically this could have introduced variation in sex induction during the selection experiment. However, females of *B. calyciflorus* respond quickly to mixis cues (Gilbert 2007) and induction of sex is commonly reported to take place at densities lower than 1 female/mL (Gilbert and Schröder 2007). Since the initial number of females was always 2.2 females/mL, we expect that sexual reproduction was initiated from the beginning of each growth period.

### Assay of local adaptation

After eight weeks of selection, once the fitness increase of the rotifer populations of the harsh environments was significant, 2 × 30 females from each replicate were isolated for each population and used in standardized fitness assays. These small subpopulations were first transferred into 30 mL of “local” or “foreign” medium to remove maternal effects. If transferred to the foreign medium the conditions were slightly reduced for the next 3 days to allow for physiological acclimatization by adding 5 g/L salt (SM) and 180,000 cells/mL (FM). After 3 days, 20 females of the second generation were transferred to the full selection medium. After one additional day, twelve newly hatched females (*n* = 60 per treatment) of the third generation were placed individually into 2 mL of full selection medium in 24-well-plates and were allowed to reproduce and build up a subpopulation for 4 days. Finally, these subpopulations were fixed with Lugol's solution and population density and population growth rates were estimated by microscopic counting.

### Statistical analysis

Statistical analyses for the repeated measures data from the selection experiment were done in R v. 2.12.1 (R Development Core Team 2010). As described by (Logan 2010) and (Zuur et al. 2009), we used linear mixed effects models (LME) using the “nlme” package (Pinheiro et al. 2012) to search for differences between treatments for fitness (growth rates), ratio of diapausing eggs per female, and ratio of males per female. We used the time “Day” as covariate and the “Treatment” (CP, SP and FP) as fixed effect with an interaction term, applying the REML method. Random effects were included for “Replicate” nested within “Treatment” (combined into ID), and for “Day”. If improving the reliability of the models, we added spatial correlation structures and corrected for heterogeneity using different variance structures provided by Logan (2010) and Zuur et al. (2009). The process of model selection was based on the Akaike Information Criteria (Logan 2010). Model validation was done graphically using diagnostic plots. The LME over “Day” for fitness was calculated after exponentially transformation without any additional structures. Since in the global dataset the short time period produced a very high variance, presumably because of an extended lag phase after the long period, analyses were restricted to data points of the long period (4 days interval of the selection and adaptation experiment). In addition, we compared LMEs using “Day” as *factor* and as *ordered factor* to test for non-linear trends and for factor level significances (see Data S1). A generalized additive mixed effects model (GAMM) from the “mgcv” package (Woods 2012) was additionally used to test for “smoothers” producing a significant slope, using eight knots and cubic regression with the same covariates, fixed and random effects. The LME on “Day” for the ratio of diapausing eggs per female was calculated using a *corCompSymm* correlation structure. The LME on “Day” for the ratio of males per female was calculated using a *varExp* structure for improving homogeneity. For more details on single models applied to fitness, diapausing eggs and males, information is given in the Data S1.

Spontaneous hatching of diapausing eggs was analysed using a generalized linear mixed effects model (GLMM) of the binomial family using the “lme4” package (Bates et al. 2009), comparing the percentage of eggs that hatched in the two different treatments. “Days” and “Treatment” (“Light” or “Dark”) were used as covariate and fixed effect respectively, while random effects were assumed over an “ID” with “Well” nested in “Treatment” and on “Days”.

For the local adaptation test, the assumptions of normality, linearity and homogeneity were violated and could not be corrected using any transformation. Therefore, mean values for each replicate population, which were normally distributed, were used to analyse the difference in interaction between population and medium. This was done using a two-factorial ANOVA to test for the “home” versus “away” criterion. For the full data set, the treatments were compared using a Kruskal–Wallis test. Population densities for the two media (SM and FM) were *a priori* compared with two Mann–Whitney *U* tests to test for the “local” versus “foreign” criterion.

## Results

### Spontaneous hatching rate

Diapausing eggs fertilized in the lake “Egelsee” population hatched spontaneously under laboratory conditions. The hatching rate differed significantly between the “Light” and the “Dark” treatment (Table S1). In the “Dark” treatment the diapausing eggs started to hatch immediately after the incubation was initiated (in total 70.85%), while in the “Light” treatment diapausing eggs hatched with a delay of about 3 days (Fig. S1, in total 63.39%). Altough the total level of hatching was indicated to be significantly lower in the GLMM for the “Light” treatment a hatching rate of more than 50% was considered to be suitable for our experiment.

### Divergent selection

Here we present the results of the process of selection over time. The test of local adaptation will be presented in the next section. The mean growth rates of rotifers after the first 4 days in normal-COMBO medium were 1.00 per day (± 1 SE = 0.062) starting from a density of 2 females/mL. In the food-limited population the cell numbers of the algae remained at 150,000 cell/mL for 4 days, and the rotifer population growth rate was lower (mean growth rate 0.68 per day, ± 1 SE = 0.014). Growth rates were also lower in the high-salt population (mean growth rate 0.33 per day, ± 1 SE = 0.055). Algae grew positively for the days between the transfers (algae cell density in SM increased up to 1 million cells/mL). Fitness of the harsh treatments was significantly reduced in both treatments compared to the normal-COMBO population (NCP) in an ANOVA with planned comparisons after log transformation (CP vs. FP df = 1, 12, *F* = 7.3756, *P* = 0.01857; CP vs. SP df = 1, 12, *F* = 62.0483, *P* < 0.0001). This confirmed that our experimental conditions really reduced the fitness of the rotifers, leaving the opportunity for adaptation in these populations.

**Figure 1:**
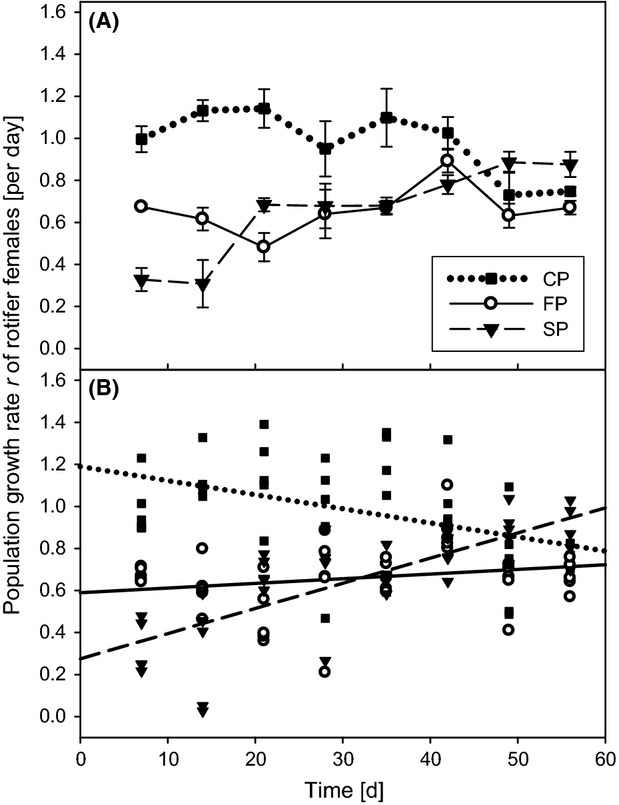
Change of fitness of rotifers in the selection experiment over time. (A) population growth rates *r* (mean values and ± 1 standard errors bars; *n* = 5); (B) best-fit linear model (each symbol represents a replicate population). Slopes of both treatments differed significantly from the benign conditions (see Table 1), indicating a stronger fitness increase over time. CP: normal-COMBO populations; SP: high-salt populations; FP: food-limited populations.

During the 56 days period of selection and adaptation, fitness of females diverged significantly in the populations (Fig. 1A). The fitness level of females in the high-salt medium environment and of the food-limited environment increased over time relative to the beginning in comparison to the rotifers cultivated in the normal-COMBO environment, suggesting adaptation to these environments (Fig. 1B). The increase in fitness in the food-limited population was positive, but reduced in comparison to the high-salt population. In the normal-COMBO populations fitness seemed to decrease over time. We found predominantly linear trends (“*ordered factor* Day” see Data S1), suggesting a linear model as mathematical basis (Table S2). There was a very weak cubic trend in the food-limited population (Table S2), however a cubic model did not improve the AIC criteria. In the linear mixed effects model (LME), which we applied over time by using “Day”, we found a fitness increase for the food-limited population, (Table 1, Day × FitnessFP; Estimate = 0.0089, df = 102; *t* = 3.4895; *P* = 0.0007) in comparison to the normal-COMBO populations. For the high-salt population the value characterizing the slope was also significantly higher (Table 1, Estimate = 0.0186, df = 102; *t* = 7.2751; *P* < 0.0000) compared to normal-COMBO populations. However, some of this increase in fitness might be significant as the normal-COMBO populations significantly decreased (Table 1, Estimate = −0.0067, df = 102; *t* = −3.7036; *P* = 0.0003). The model validation plot for the LME using “Day” did not give any reason for concern about distribution of the residuals (Fig. S2). We tested for a significant effect of the “*factor* Day” to test for changes at particular time points. Those analyses showed significant differences for most of the final days in the high-salt population (Table S3). The GAMM-smoothers indicated results comparable to findings from the LME. The slope of the “smoother” was only significant for the high-salt population and the normal-COMBO populations (Table S4). Examination of the fitted “smoothers” by plotting (Fig. S3) showed that the high-salt population “smoother” increased linearly. In the food-limited population fitness remained constant over time producing a wave pattern, while in the normal-COMBO population fitness decreased. Finally these results propose that there was no real increase in fitness over time relative to the beginning in the food-limited population and that this population improved in fitness only in comparison to the normal COMBO population.

**Table 1:** Results of the linear mixed effects model on female fitness over time. There was no correlation structure and no improvement for heterogeneity used. Data (*n* = 5) were exponentially transformed and “Day” was used as fixed effect. The initial fitness level was lower in both harsh environments indicated by the lower intercepts. Fitness change over time of both harsh populations, represented by the slope, increased significantly over time compared to the normal-COMBO populations. In the model FitnessSP/FP is the comparison between high-salt and the food-limited populations to normal-COMBO populations. The interaction with “Day” gives the slope, or increase over time relative to the beginning, in relation to the slope in the normal-COMBO populations. The intercept gives information about if the level of fitness

	Value	SE	df	*t*-value	*P*-value
FitnessCP	1.1891	0.0641	102	18.5655	0.0000***
Day × FitnessCP	−0.0067	0.0018	102	−3.7036	0.0003***
FitnessFP	−0.5999	0.0906	12	−6.6230	0.0000***
FitnessSP	−0.9125	0.0906	12	−10.0737	0.0000***
Day × FitnessFP	0.0089	0.0026	102	3.4895	0.0007***
Day × FitnessSP	0.0186	0.0026	102	7.2751	0.0000***

Fitness of rotifer females: growth rates.

SP, high-salt population; FP, food-limited population; CP, Normal-COMBO populations.

***0, **0.001, *0.01, 0.05, 0.1, 1.

**Figure 2:**
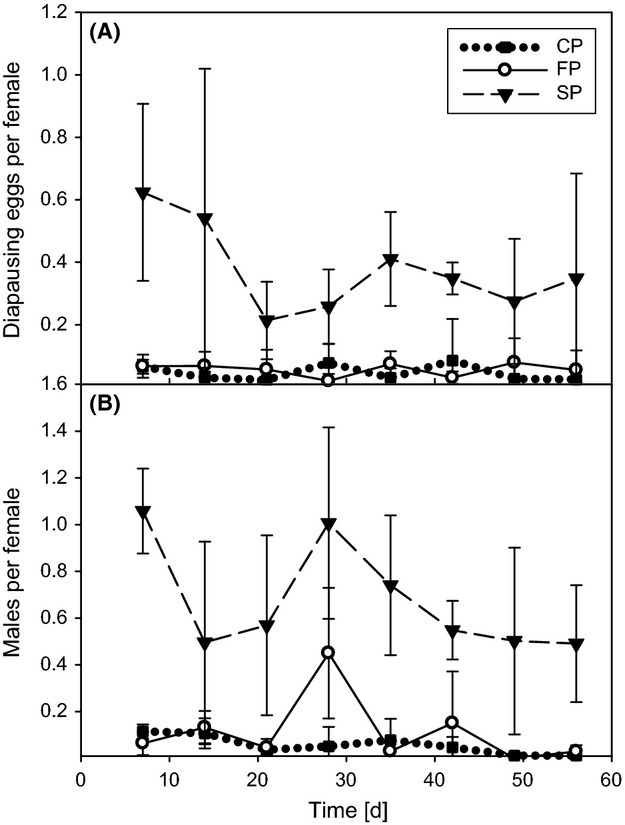
The ratios of sexual offspring during the selection experiment. (A) Diapausing eggs per female; (B) Males per female (mean values and ± 1 standard error; *n* = 5). In the linear mixed effects models (regression lines not shown) the ratios of sexual offspring of the high-salt populations differed significantly (Table 2 and Table 3). CP: normal-COMBO Conditions; SP: high-salt population; FP: food-limited population.

Over the duration of the experiment the ratio of diapausing eggs per female (Fig. 2A) and the ratio of males per female remained mostly constant (Fig. 2B). There was an increase in males shortly before day 30 for which we have no obvious explanation. The average ratios for diapausing eggs per female were 0.38 and for males per female 0.68 in the high-salt population. In the food-limited population these ratios were very low (FP: 0.06 for diapausing eggs and 0.11 for males), which was expected because sex is known to be repressed during food-limitation in rotifers (Gilbert 2010).

In the LME on “Day” for the ratio of diapausing eggs per female the diagnostic plot indicated small problems with heterogeneity. To test the reliability of the model, we used an *F*-test to compare the variances in the LME and found no significant deviation from homogeneity (*F* = 1.3433; df = 59; *P* = 0.2599). The ratio of diapausing eggs per female was greater than zero in the normal-COMBO population, but did not change over time. In the food-limited population there was no difference (Table 2). In the high-salt population the ratio was significantly higher (Estimate : 0.4719; df = 12; *t* = 3.4988; *P* = 0.0044) indicating a higher level of diapausing eggs per female compared to the normal-COMBO population.

**Table 2:** Results of the linear mixed effects model on the ratio of diapausing eggs per female. A correlation structure *corCompSymm* was used to improve the model fit, but no correction for heterogeneity was necessary. Data were not transformed (*n* = 5) and “Day” was used as fixed effect. The ratio of diapausing eggs per female produced by the high-salt population was significantly higher compared to the food-limited population. The ratios did not change over time (Fig. 2A). In the model DiEgg/Fem is the comparison between high-salt and food-limited populations to normal-COMCO populations

	Estimate	SE	df	*t*-Value	*P*-value
DiEggs/FemCP	0.5522	0.0954	102	5.7899	0.0000 ***
Day × DiEggs/FemCP	−0.0002	0.0023	102	−0.1044	0.9170
Di Eggs/FernFP	0.0053	0.1349	12	0.0392	0.9694
DiEggs/FemSP	0.4719	0.1349	12	3.4988	0.0044**
Day × Di Eggs/FernFP	0.0002	0.0033	102	0.0523	0.9584
Day × DiEggs/FemSP	−0.0044	0.0033	102	−1.3320	0.1858

DiEggs/Fem = ratio of diapausing eggs per female.

SP, high-salt population; FP, food-limited population; CP, Normal-COMBO populations.

***0, **0.001, *0.01, 0.05, 0.1, 1.

The ratio of males per female slightly decreased over time in the normal-COMBO populations despite there was an increase shortly before day 30 (Table 3; Estimate = −0.0018; df = 102; *t* = −3.1128; *P* = 0.0024). The ratio was significantly higher for the high-salt population (Estimate = 0.8022; df = 12; *t* = 6.9913; *P* < 0.0000) indicating a higher level of males per female compared to the normal-COMBO population and a decrease over time at a similar rate. The slope and the ratio in the food-limited population were statistically not different from the normal-COMBO population (Table 3).

**Table 3:** Results of the linear mixed effects model on the ratio of males per female. There was no correlation structure necessary, but a *varExp* correction for heterogeneity was improving the model. The data (*n* = 5) were not transformed and “Day” was used as fixed effect. The ratios of males per female were significantly higher in the high-salt population. Over time the ratios declined in both treatments (Fig. 2B). In the model Ma/Fem is the comparison between high-salt and food-limited populations to normal-COMCO populations

	Estimate	SE	df	*t*-Value	*P*-value
Ma/FemCP	0.1151	0.0244	102	4.7146	0.0000***
Day × Ma/FemCP	−0.0018	0.0006	102	−3.1128	0.0024**
Ma/FemFP	0.0640	0.0717	12	0.8932	0.3893
Ma/FemSP	0.8022	0.1147	12	6.9913	0.0000***
Day × Ma/FemFP	−0.0002	0.0019	102	−0.0972	0.9227
Day × Ma/FemSP	−0.0057	0.0034	102	−1.6625	0.0995

Ma/Fem = ratio of males per female.

SP, high-salt population; FP, food-limited population; CP, Normal-COMBO populations.

***0, **0.001, *0.01, 0.05, 0.1, 1.

### Assay of local adaptation

**Figure 3:**
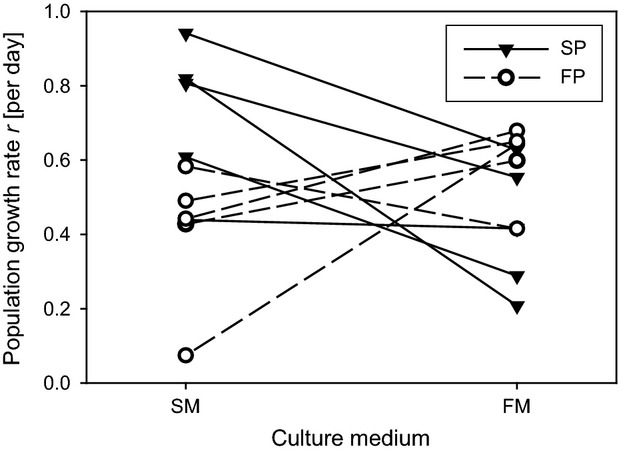
Mean population fitness in the test for local adaptation. Fitness of each “local” population was compared to the fitness of the “foreign” population and at “home” versus “away” conditions (*n* = 24 per replicate). Lines connect identical populations in different environments. There was a significant crossing pattern as proposed by Kawecki and Ebert (2004) pointing to local adaptation (Table 4). SM: high-salt medium; FM: food-limited medium. SP: high-salt population; FP: food-limited population.

We found a clear pattern of local adaptation after the divergent selection and adaptation experiment (Fig. 3 and Table 4). Populations from the high-salt medium had a higher fitness compared to food-limited populations when cultivated in the high-salt medium. Furthermore, the high-salt population had a higher fitness in the high-salt medium compared to the fitness obtained in the food-limited medium. For the food-limited population we detected a similar pattern: this population was more fit at home than in the away environment while it had a slightly higher fitness compared to the high-salt adapted population when cultivated in food-limited medium.

**Table 4:** Results of the ANOVA comparing the interaction between population and medium for the test of local adaptation. Fitness of populations was compared for the “home versus “away” conditions (*n* = 24 per replicate). The significant interaction term indicates that the slopes for both populations were crossing and not parallel (Fig. 3). The terms “Population” and “Medium” represent high-salt and food-limited population and high-salt medium and food-limited medium respectively

	df	Sum SQ	Mean Sq	*F*-value	Pr(>*F*)
Population	1	0.0259	0.0259	0.8755	0.3633
Medium	1	0.0146	0.0146	0.4925	0.4929
Population: Medium	1	0.3125	0.3125	10.5556	0.0050**
Residuals	16	0.4737	0.0296		

**0.001.

To investigate the local versus foreign criterion we compared our data from the assay of local adaptation using a non-parametric test, since assumptions for a parametric test were violated. In a Kruskal-Wallis we compared the raw data on growth rates of the assay, which revealed significant differences for the treatments within the test of local adaptation (Kruskal–Wallis χ^2^ = 36.955, df = 3, *P* < 0.0001). Next we used two Mann–Whitney *U* tests exploring local versus foreign populations (FM: *W* = 2407.5, *P* = 0.001334; SM: *W* = 2660.5, *P* < 0.0001). This indicated that there was a significant difference between the mean values, when comparing local versus foreign.

The count data of the assay of local adaptation violated the assumption of normality and linearity as stated above, thus we looked on the home versus away criterion using the interaction in an ANOVA based on the mean values of each replicate population. The interaction term in the ANOVA based on the means between population and medium was significant (Table 4, F = 10.5556; df = 1, 16; *P* = 0.0050), indicating that between populations slopes were crossing and not parallel and confirming the home versus away criterion.

In total the results for the food-limited population were less obvious. One subpopulation of the food-limited population had a higher fitness in the foreign environment. The food-limited population was at home only slightly fitter than the high-salt population in the food-limited medium. In total, there was a crossing pattern that local populations were superior over foreign populations and additionally less fit in the away habitat compared with the home conditions, pointing to local adaptation (Fig. 3), but with obvious differences for the habitats. For the high-salt population the pattern of adaptation was much more obvious. These results confirm that fitness for a given population in its local environment was higher than of a population from the foreign habitat, but less obvious for the food-limited population. Thus, local adaptation was detected.

The number of generations necessary to detect local adaptation can be estimated by the growth rates. During the selection experiment the mean asexual growth rate over all treatments was 0.763 per day. We followed the experiment for 56 days, which corresponds to approximately 43 asexual generations. We estimate a value of one sexual generation per week, because diapausing eggs were produced shortly before the transfer to fresh medium (3–4 days) and hatched with a delay of 3–4 days without the dark/cold trigger, which results in eight sexual generations.

## Discussion

In this study we provide evidence that experimental populations of the rotifer *B. calyciflorus* rapidly develop local adaptation to high-salt and low-food environments under a selection regime that explicitly favours fast population growth. These results are comparable to results obtained for yeast (Gray and Goddard 2012a) and show that rotifers can be used as a model to test for local adaptation after laboratory natural selection. The study of Becks and Agrawal (2010) tested for local adaptation in two homogenous environments using rotifers, but could confirm local adaptation only partly as they could show the local versus foreign criterion for one side only. Thus our study fills this gap by providing data that local adaptation due to LNS can be rapidly observed in rotifers.

The level of adaptation seemed different for the two environments: it seemed to be higher in the high-salt than in the food-limited environment (Fig. 1). This observation was supported both by the data of the selection experiment itself (a larger increase of mean population growth over time in the salt-populations) as well as in a subsequent test for local adaptation. Both local populations had a higher fitness than the foreign populations and were more fit at “home” than in the “away” environment (Table 4 and Fig. 3). Populations raised in their local habitat had on average a 38% higher fitness than populations raised in the foreign habitat, which is comparable to the 45% value reported by Hereford (2009). The higher level of adaptation in the high-salt populations during the selection phase, measured relative to the normal-COMBO populations, were accompanied by higher levels of sex (i.e., males per female, diapausing eggs per female; Fig. 2). According to our results of the spontaneous hatching rate of diapausing eggs, we estimated that about 43 asexual and eight sexual generations passed during the selection and adaptation phase.

The decrease of fitness in the control population over time is difficult to explain. One might expect that a population from the field introduced to new conditions (e.g., our control environment) would also undergo adaptation, thereby increasing in fitness, a process called evolutionary domestication (Simões et al. 2007). Potentially, our normal-COMBO populations over-exploited their resources during the times between transfers in the selection experiment and thereafter ran into starvation. In that case, the transfer period might have been too long for the control. However, this time period was necessary for the two harsh conditions, where the population growth rates were reduced. In experimental yeast populations the growth rate *r* increased, but the yield in biomass declined (Jasmin et al. 2012). In our normal-COMBO populations rotifers might have been selected for starvation tolerance rather than high growth rates, but this is highly speculative. Because of these uncertainties we did not include the normal-COMBO populations in the test for local adaptation (which would have been a better design) and used this “benign” control only to detect a fitness increase for the time of selection in the treatments. Thus, the result of higher adaptation in the high-salt population cannot quantitatively be confirmed, which would be possible if these comparisons would be done. As there was something going on since the normal-COMBO populations probably also evolved during the selection experiment, the data from the normal-COMBO population might be regarded as a third treatments rather than a control. However, we propose that the observation of fitness decrease under normal COMBO conditions does not influence our interpretation of local adaptation in the two harsh conditions, which was the main focus of your study.

Our results suggest that the process of selection and adaptation can be influenced by the type of habitat, as we found a less pronounced increase in fitness in the food-limited population compared to the high-salt population, having a much higher slope in the LME. There are several possible explanations for this difference. First, our experimental population might have been at a different distance from the final adaptive peak in the two environments (Poelwijk et al. 2007). This could be due to a lack of relevant genetic variation in the low-food environment (Barrett and Schluter 2008). We isolated our rotifers from the field, which at least periodically should select for low food conditions and introduce adaptations to low-food conditions into the population. However, similar to that, adaptation to increased salinity should occur in populations that inhabit ephemeral water bodies. However, we detected adaptation only in the high-salt population but not in the food-limited population. The fitness of food-limited populations was 33% lower compared to the normal-COMBO environment, giving some room for adaptation. As there were no more selection pressures than food-limitation, one should expect that the food-limited populations should at least partially increase in fitness due to adaptation of metabolic pathways. Other studies found rapid adaptation to starvation of *Drosophila melanogaster* within a few generations by metabolic and behavioural changes (Schwasinger-Schmidt et al. 2012), which clearly demonstrates that organisms can increase their fitness under such conditions. A second explanation for the difference in adaptation to the two environments relates to the rate of sexual reproduction. In our experimental populations sexual rates were increased in the high-salt populations compared to the low-food populations. This observation is in agreement with the idea that sex might increase adaptation to new environmental conditions (Colegrave 2002; Kaltz and Bell 2002; Goddard et al. 2005; Becks and Agrawal 2010, 2012; Dudycha et al. 2013).

Our data on male and diapausing egg ratios suggest that rates of sex were elevated in the high-salt environment already from the beginning of the experiment and remained relatively high throughout (Fig. 2). Thus, we suggest that the high-salt environment either acted as a trigger for sex or, alternatively, that the normal-COMBO and low-food environment suppressed sex. Overall sex induction in *Brachionus* is mediated by a density-dependent protein, which is excreted by the females in a process analogous to *quorum sensing* (Stelzer and Snell 2003; Snell et al. 2006; Kubanek and Snell 2008). However there are several environmental and genetic factors that modulate the response to this chemical (Gilbert 2003b). For example, extreme conditions near the physiological limits of rotifers tend to suppress sexual reproduction (Snell 1986). Furthermore, the production and viability of diapausing eggs depends on the availability of food (Gilbert 2010). Thus food limitation might have partly repressed sexual reproduction in our experiment. As for the second hypothesis, we are not aware of any study showing that high-salt environments elicit elevated rates of sexual reproduction in *B. calyciflorus*, but we cannot exclude this possibility.

In addition to these phenotypic responses to environmental factors, rates of sexual reproduction might themselves evolve during selection experiments. For example, Becks and Agrawal (2010, 2012) showed that sex in their rotifer cultures was evolving towards higher levels during adaptation. Other authors (Smith and Snell 2012) recently found a rapid evolutionary increase of sex in rotifers under temporary conditions (i.e., short hydroperiod duration). Fussmann et al. (2003) and Stelzer (2011) demonstrated a rapid evolutionary loss of sex in benign environments in rotifer cultures (Fussmann et al. 2003; Stelzer 2011). Such evolutionary responses can be confirmed by standardized assays that measure the propensity for sex of individual clones isolated from the evolving populations at different times during the selection experiment. Since we did not conduct such assays we cannot rule out the possibility that such evolutionary responses occurred later in our experiment. However two observations suggest that a direct environmental influence was at least more important than an evolutionary response: First, our base population exhibited different sexual rates in high-salt versus low-food environments already at the beginning of the experiment. Second, our indicators of sex in the evolving populations (males per females, diapausing eggs per female) did not suggest that sexual rates changed considerably or directionally during the experiment (see Fig. 2). The data presented in Fig. 2 propose that sex did not evolve over time by selecting clones with high sexual propensity. The only change was observed for males per female, but this was very small and cannot be used to prove an evolutionary change. Thus, most likely food-limitation has suppressed higher sex levels in our food-limited clones.

We did not measure fitness of both treatment populations at the beginning of the experiment, since all populations consisted of the same 83 clones. Therefore, it is highly unlikely to see the observed pattern of local adaptation before the selection took place. Thus, we can assume that there was no trade-off for the populations at the start of the experiment. If the rotifers would have been perfectly adapted to one of the experimental conditions, because of similar selection in the field, we would not expect to see one population performing better in the home habitat after selection compared to the away conditions. It is possible, that the populations performed a little better in one environment than in the other as suggested by Fig. 1 when isolated from the field. But, after selection, populations had a higher fitness in their home habitat than in the away habitat. This can only be explained by local adaptation.

Our results support the idea that two different stressful environments (high-salt, low-food) might produce different levels of adaptation. The intensity of selection can be very important for adaptation (Robertson 1960; Jones et al. 1968; Rumball and Rae 1968; Frankham 1977). The effect of selection is predicted to first increase with intensity, but only decrease at very high intensity (Bell 2008). We used two stressful habitats in our study since we could not take an increase in fitness under benign conditions, such as normal-COMBO medium, for granted (Goddard et al. 2005; Gray and Goddard 2012b). For example, studies on *Saccharomyces cerevisiae* have shown that fitness increased over time in harsh environments but remained essentially unchanged in benign environments (Goddard et al. 2005; Gray and Goddard 2012b). In this respect it is interesting that the initial fitness of our high-salt populations was significantly lower than that of the low-food populations (see in Table 1), suggesting that the high-salt conditions were harsher than the food-limitation, but the fitness gain during adaptation was higher in the high-salt populations.

In a study by Becks and Agrawal (2012), two lines of *B. calyciflorus,* which were already adapted to harsh or benign conditions were transferred to the respective “away” environment while control populations stayed in the environment to which they were adapted. The authors found an initial drop in fitness of populations in the “away” environment followed by a recovery within approximately 50 days. Additionally, the level of sex increased during adaptation and sexual offspring had a higher fitness than asexual offspring (Becks and Agrawal 2012). In the study of Becks and Agrawal (2012) changes in fitness during adaptation were measured in separate assays which differed from the conditions experienced by the adapting populations: While the adapting populations were maintained under a serial dilution regime involving replacement of only 10% of the culture volume every other day, the separate assays quantified lifetime reproductive output of individually cultured females. One might argue that the serial dilution regime involves more or less stationary growth (i.e., a growth rate of approximately 0.05 per day, which is an extremely small value compared to the maximum growth rates that can be reached by *B. calyciflorus*) and very high population densities. By contrast the separate assays, which were conducted under benign conditions (individuals cultured with daily transfers to fresh culture medium), resemble exponential growth conditions in an “empty” habitat. In our study we tried to avoid such incongruity by providing very similar conditions in the adapting populations and assay individuals, respectively (see Materials and Methods). Notwithstanding these methodological differences, the speed of adaptation and fitness gains in our populations adapting to the high-salt environment were quite comparable to those reported by Becks and Agrawal (2012).

Our data support the view that not a general fitness genotype evolved being superior under all laboratory conditions, but that at least two (probably several) different specialized genotypes locally adapted. However this is not an ultimate prove- a statement would require a detailed analysis of the evolved populations using molecular markers. The observation of local adaptation is hard to explain with the idea of a general fitness genotype, because it is not clear why this genotype should have a lower fitness in an away habitat. Even harder to explain is why there should be a foreign depression in case there is a general fitness genotype. Thus, we expect that at least two different genotypes were selected for the high-salt and food-limited environment, respectively. Local adaptation can be estimated for two different habitats (Kawecki and Ebert 2004; Konijnendijk et al. 2013). This can be two harsh, or a harsh and a benign habitat, but ultimately the habitats have to be different. For future research it would be interesting to evaluate local adaptation in rotifers under benign conditions. Finally, we think to test for local adaptation would be a good alternative in case cryopreservation is not suitable.

## Supporting Information

**Data S1.** Materials and methods.

**Figure S1.** Percentage of emerged diapausing eggs in the spontaneous hatching experiment.

**Figure S2.** Diagnostic plot of the linear mixed effects model for “Day” during the time of adaptation for the rotifer populations.

**Figure S3.** Smoothers for the fitness of females over the time of adaptation extracted from the generalized additive mixed effects model.

**Table S1.** Results of the generalized linear mixed effects model on percentage of hatched diapausing eggs.

**Table S2.** The change of fitness for females over time was explored for linear or polynomial trends in the linear mixed effects model with “*ordered factor* Day”.

**Table S3.** The change of fitness for females over time was explored using “*factor* Day” for fitness of females.

**Table S4.** The generalized additive mixed effect model testing for a significant slope in fitness of females.
