# Supplementary material for: Patterns and dynamics of rapid local adaptation and sex in varying habitat types in rotifers
Source: Ecol Evol. 2013 Oct 1;3(12):4253–64. doi: 10.1002/ece3.781 (PMC3853569; doi:10.1002/ece3.781)
Supplement: Supplementary file 1 [file ece30003-4253-SD1.doc]

# Supporting Information

**Patterns and dynamics of rapid local adaptation and sex in varying habitat types in rotifers**

**Thomas Scheuerl, Claus-Peter Stelzer.**

# Material and Methods

## The study system

As proposed by Fussman (2011) we used rotifers (the Monogonont rotifer *Brachionus calyciflorus*) as model organisms, because they can be easily cultured, reproduce quickly, and occur at high levels of clonal genetic diversity in nature. Monogonont rotifers are cyclical parthenogens with haploid dwarf males, which do not feed any more (Fussmann, 2011). Females normally reproduce by ameiotic parthenogenesis, but initiate sporadic sexual episodes, producing sexual and asexual daughters (Nogrady, 1993). Induction of sexuality is mainly density dependent (Gilbert, 2003; Stelzer & Snell, 2003; Schröder, 2005). At high population densities a chemical cue induces the production of sexual females (Stelzer & Snell, 2003; Snell & Stelzer, 2005). The oocytes of these sexual females undergo meiosis and develop into haploid males (if not fertilized), or diploid diapausing eggs (if fertilized).

## Isolation of diapausing eggs

Subsamples of 10 g (wet weight) were taken from a sediment sample collected from lake `Egelsee` three days before isolation was started. Each subsample was resuspended in 1.75 mol L^-1^ sucrose solution in 45 ml centrifuge tubes and centrifuged at 10 g for 5 min. The supernatant was washed through a 500-µm Nytal mesh and collected with a 30-µm mesh. The former mesh removed large material, while the diapausing eggs were retained on the 30-µm mesh.

## Stem cultures

Stem cultures of rotifers and algae were maintained at 21°C and continuous illumination was provided with daylight fluorescent bulbs (30–40 µEinstein m^-2^ s^-1^ for rotifers; 200 µEinstein m^-2^ s^-1^ for algae). Clonal cultures of rotifers were re-inoculated twice per week by transferring 6-7 asexually reproducing females to fresh culture medium (5 ml) provided in six-well-plates. Algae were supplied at *ad libitum* concentrations (~400,000 cells ml^-1^).

## Spontaneous hatching rate

Two asexual females from each of 70 different clones were inoculated together in 2L food suspension. After 3 days, when production of diapausing eggs started, the population was concentrated using a 30-µm mesh. All diapausing eggs were collected and placed individually in a well of 48-well-plates. After removal of diapausing eggs, the population was re-incubated for another 24 hours. All diapausing eggs collected at a particular day were divided into two treatments, a `Dark` and a `Light` treatment.

## Divergent selection and adaptation

The concentrations of salt and nitrogen necessary to reduce the growth rate within three and four days were experimentally determined in series of pre-experiments. For this, we used 2 clones isolated from lake `Egelsee`. For the present experiment we isolated 112 new clones from the lake and cultured them individually. Unfortunately only 83 clones survived for two weeks until the experiment started. Thus, there seemed to be a high variance in fitness in these rotifer clones. The populations of the food-limited medium (FM) and high-salt medium (SM) treatments were acclimatized to the constraining conditions by a reduced step for the first three days (FM: 10 µMol N & 0.03 OD and SM: 5 g L^-1^). Food algae for the selection experiment were grown separately in 2 L borosilicate bottles. Algae were concentrated by centrifugation at 3000 rpm for 10 minutes and diluted to the desired food concentration and in the desired medium. At the end of each growth period, the numbers of females, diapausing eggs and males were determined by counting subsamples fixed with Lugol`s solution using inverted microscopy at 200-fold magnification. The appropriate volume for the next period was filtered using a 30-µm mesh (retaining females, diapausing eggs and partly males) and washed into the newly prepared bottle. All material was rinsed with 70 % alcohol and de-ionized water prior use in the next transfer. After successful transfer, another subsample of living females was counted.

## Additional statistical information

In this section additional statistical details for the linear mixed effects models and additive mixed effects models for the analysis of the fitness data of adapting rotifer females and for ratios of males per female and diapausing eggs per female, as well as for the sex ratios are given. This part involves details on used correlation structures and applied correction structures for heterogeneity as provided by other authors (Zuu*r et a*l., 2009; Logan, 2010). When `Day` was used under different levels in the LME, the output of the model gave results as follows: i) `Day` used as *ordered factor* analysed linear and polynomial trends in the data set, ii) `Day` used as *integer* displayed the overall slope differences of the model and iii) `Day` used as *factor* revealed statistical differences between each factor level of `Day`.

The Kolmogorov Smirnov test (normality) and the Bartlett test (homogeneity) were used for more precise selection between similar transformations. For the GAMM model of fitness, no tested correlation structure improved the model. Model validation did not give any reasons for concern. Heterogenity was corrected with *varExp* function. Exploration of the global data set (long period combined with short period) did not greatly change the results (data not shown).

# References

Fussmann, G.F. 2011. Rotifers: excellent subjects for the study of macro- and microevolutionary change. *Hydrobiologia* **662**: 11–18.

Gilbert, J.J. 2003. Specificity of crowding response that induces sexuality in the rotifer *Brachionus*. *Limnol. and Oceanog.* **48**: 1297–1303.

Logan, M. 2010. *Biostatistical Design and Analysis Using R. A practical guide*, 1. Auflage. John Wiley & Sons.

Nogrady, T. 1993. *Rotifera 1 Biology Ecology and Systematics*. Balogh Scientific Books.

Schröder, T. 2005. Diapause in monogonont rotifers. *Hydrobiologia* **546**: 291–306.

Snell, T.W. & Stelzer, C.-P. 2005. Removal of surface glycoproteins and transfer among *Brachionus species*. *Hydrobiologia* **546**: 267–274.

Stelzer, C.-P. & Snell, T.W. 2003. Induction of sexual reproduction in *Brachionus plicatilis* (Monogononta, Rotifera) by a density-dependent chemical cue. *Limnol. and Oceanog.* **48**: 939–943.

Zuur, A.F., Ieno, E.N., Walker, N., Saveliev, A.A. & Smith, G.M. 2009. *Mixed effects models and extensions in ecology with R*, 1st Edition. Springer.

# Figures

**Fig. S1. Percentage of emerged diapausing eggs in the spontaneous hatching experiment.** In the `Light` treatment diapausing eggs were directly incubated after production. In the `Dark` treatment diapausing eggs were stored at 8°C for two weeks prior to incubation. Despite a lower number of females hatched in the `Light` treatment than in the `Dark` treatment (63.4 % compared too 70.9 %), a spontaneous development higher than 50 % was considered appropriate for our experiment.

**Fig. S2**. **Diagnostic plot of the linear mixed effects model for `Day` during the time of adaptation for the rotifer populations.** The plot was extracted from the R environment showing the distribution of the residuals. There was no reason of concern as there was no obvious pattern observed.

**Fig. S3.** **`Smoothers` for the fitness of females over the time of adaptation extracted from the generalized additive mixed effects model.** The slope of the `smoothers` for the high-salt population was significant. There was no difference to zero detected in the food-limited population. Homogeneity was improved with *varExp* function. No correlation structure improved the model. Eight knots and cubic regression was used to create the smoothers. Broken lines indicate the 95% confidence interval. Control refers to the normal-COMBO populations; SP refers to the high-salt populations; FP refers to the food-limited populations.

# Tables

**Table S1.** **Results of the generalized linear mixed effects model on percentage of hatched diapausing eggs.** The model was calculated on a binomial distribution with repeated measures of hatched diapausing eggs. In the Dark treatment the ratio of hatching significantly increased. The level of hatching in the Light treatment was lower, but increased later on with a higher slope.

**Table S2.** **The change of fitness for females over time was explored for linear or polynomial trends in the linear mixed effects model with `*ordered factor* Day`.** There was no correlation structure used. The *varExp* method slightly improved the model by correcting weak heterogeneity structures. Trends were either linear (SP) or cubic (FP), while quadratic trends were never detected. A cubic LME did not improve the AIC during model selection. CP: normal-COMBO populations; SP: high-salt populations; FP: food-limited populations.

**Table S3.** **The change of fitness for females over time was explored using `*factor* Day` for fitness of females.** There was no correlation structure used. A *varExp* Structure corrected for heterogeneity. In the high-salt population there was a constant increase in fitness, while this was only detected for a short time in the food-limited populations. CP: normal-COMBO populations; SP: high-salt populations; FP: food-limited populations.

**Table S4. The generalized additive mixed effect model testing for a significant slope in fitness of females.** The model was calculated without correlation structure with 8 knots and cubic regression. Homogeneity was improved with *varExp* function. The smooth term was only significant in the high-salt populations (see Fig. S3). The results are in agreement with Table 1. CP: normal-COMBO populations; SP: high-salt populations; FP: food-limited populations.
